# A Two-Stage Method to Detect the Sex Ratio of Hemp Ducks Based on Object Detection and Classification Networks

**DOI:** 10.3390/ani12091177

**Published:** 2022-05-04

**Authors:** Xingze Zheng, Feiyi Li, Bin Lin, Donghang Xie, Yang Liu, Kailin Jiang, Xinyao Gong, Hongbo Jiang, Ran Peng, Xuliang Duan

**Affiliations:** 1 College of Information Engineering, Sichuan Agricultural University, Ya’an 625000, China; 202005739@stu.sicau.edu.cn (X.Z.); 202005483@stu.sicau.edu.cn (F.L.); 201902098@stu.sicau.edu.cn (B.L.); x_dong_hang@163.com (D.X.); 202005572@stu.sicau.edu.cn (Y.L.); 202005590@stu.sicau.edu.cn (X.G.); scaujhb@163.com (H.J.); pengran@stu.sicau.edu.cn (R.P.); 2 College of Science, Sichuan Agricultural University, Ya’an 625000, China; 202003978@stu.sicau.edu.cn

**Keywords:** farming automation, duck detection, duck sex classification, computer vision

## Abstract

**Simple Summary:**

The sex ratio of hemp ducks is considered to be a major animal welfare issue in the commercial hemp duck farming industry, but currently, it still relies on an inefficient and inaccurate manual counting method. In order to obtain an efficient and accurate way of calculating the sex ratio of ducks to solve this problem, we established the world’s first manually marked sex classification dataset for hemp ducks and used multiple deep neural network models for the target detection and sex classification of ducks, with an average accuracy of 98.68%. The evaluation of the algorithm’s performance indicates that the automation method proposed in this paper is feasible for the sex classification of ducks in the farming environment and is thus a feasible tool for sex ratio estimation.

**Abstract:**

The sex ratio is an important factor affecting the economic benefits of duck groups in the process of hemp duck breeding. However, the current manual counting method is inefficient, and the results are not always accurate. On the one hand, ducks are in constant motion, and on the other hand, the manual counting method relies on manpower; thus, it is difficult to avoid repeated and missed counts. In response to these problems, there is an urgent need for an efficient and accurate way of calculating the sex ratio of ducks to promote the farming industry. Detecting the sex ratio of ducks requires accurate counting of male ducks and female ducks. We established the world’s first manually marked sex classification dataset for hemp ducks, including 1663 images of duck groups; 17,090 images of whole, individual duck bodies; and 15,797 images of individual duck heads, which were manually captured and had sex information markers. Additionally, we used multiple deep neural network models for the target detection and sex classification of ducks. The average accuracy reached 98.68%, and with the combination of Yolov5 and VovNet_27slim, we achieved 99.29% accuracy, 98.60% F1 score, and 269.68 fps. The evaluation of the algorithm’s performance indicates that the automation method proposed in this paper is feasible for the sex classification of ducks in the farm environment, and is thus a feasible tool for sex ratio estimation.

## 1. Introduction

According to the National Bureau (2020) of Statistics of China, China has produced 76.39 million tons of pork, beef, mutton, and poultry meat, including 10.50 million tons of meat duck and 2.85 million tons of duck eggs in the past year [1]. This shows that breeding ducks plays an important role in promoting the development of the industry. At the same time, the efficient breeding of ducks also requires the help of science and technology. The communique points out that the development of the livestock industry cannot be separated from the contribution of scientific and technological progress in modern production, the contribution rate of which is between 56% and 60%, while the investment in science and technology will continue to grow during the 13th Five-Year Plan period [2].

**Figure 1:**
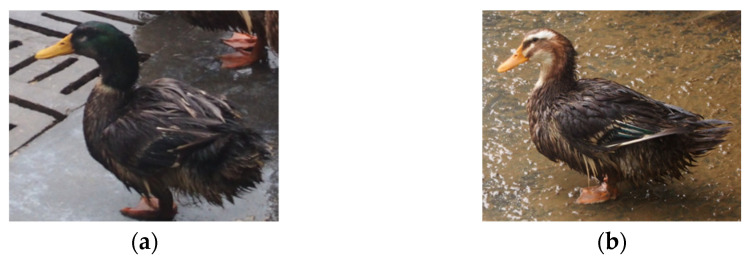
(**a**) The appearance of an average male hemp duck, while (**b**) shows the appearance of an average female hemp duck. Sex differences lead to differences in appearance.

Moderate scale farming of ducks can help to promote the economic benefits and the efficiency of farming, where an appropriate sex ratio can ensure high egg fertilization rates and fertilization quality [3]. This leads to a faster increase in the number of ducks, an expansion of the breeding scale, and ultimately, a higher economic efficiency. Conversely, if the duck sex ratio is far from the ideal ratio, fertilization rates and fertilization quality will be negatively affected, leading to a reduction in egg production and negative duck population growth. In this case, economic efficiency cannot be improved or will even be reduced. Furthermore, this ratio varies greatly depending on the species of duck, and the appropriate male to female ratio is generally 1:(2~25) for egg-type, 1:(5~8) for meat-type, and 1:(15~20) for dual-purpose-type ducks [4]. At the same time, the sex ratio has important consequences on population dynamics and viability [5]. As shown in Figure 1, the major differences between male and female hemp ducks (hereinafter referred to as ducks) are that the male hemp duck has dark green, shiny head and neck feathers, while the female one has predominantly brown freckled feathers on its head. Therefore, duck farms need to detect the number of individuals and count the number of male and female ducks in real-time. Hemp ducks are produced in the tens of millions annually. Ducks are not only fast-growing since they have a high rate of egg production but also popular because of the large number of edible parts and the tasty meat. Currently, the main method of counting the number of male and female ducks on duck farms is based on manual estimation, which has the following problems: on the one hand, workers perform repetitive counting work, which is time-consuming and labor-intensive; on the other hand, it is difficult for workers to avoid repeated and missed counts, as ducks are in constant motion.

In recent years, many novel approaches to avian identification and detection have emerged, most of which are based on sensor technology and image-processing techniques. For example, Lu Huishan et al. [6] designed a wireless sensor network-based body-temperature-monitoring system for the real-time monitoring of poultry body temperature, which can quickly detect sick and dead poultry individuals. However, wearable devices have no effective guarantee of battery life. Vroegindeweij et al. [7] proposed a simple pixel-based classification method based on spectral reflectance properties for the identification and detection of eggs, hens, housing elements, and litter using robots. Although the accuracy of this method reaches 80%, it is difficult to deploy in practice, and the accuracy hardly reaches the practical application standard. In addition, Geronimo et al. [8] used a computer vision system (CVS) and spectral information from the near-infrared (NIR) region to identify and classify chickens with wood breasts. Although the accuracy of classifying meat quality is more than 91.8%, the cost of using a spectrometer is too high and should not be applied to farms on a large scale. Geffen et al. [9] proposed a number-estimation model of laying hens based on Faster R-CNN [10] and a tracking algorithm, and its 89.6% accuracy reveals the feasibility of using computer vision. Del Valle et al. [11] proposed an unrest index for estimating the thermal comfort of the poultry model based on computer vision and image analysis, while Leroy et al. [12] proposed a computer vision method for quantifying poultry behavior. Lubich et al. [13] proposed a method for identifying, classifying, and counting poultry and eggs based on the SSD model, and the classification accuracy of eight chickens was 97%. All of the methods mentioned above focus on the behavior of individuals in groups and qualitatively identify and detect specific individuals, which is the reason why the accuracy and difficulty of deployment are not ideal.

Deep learning (DL), a branch of machine learning research, is an extremely effective method and has great potential to be used in a wide range of fields [14]. Deep learning is also showing breakthrough results in the poultry farming industry; for example, Zhuang et al. [15] used digital-image-processing techniques and deep learning to identify diseased broilers in a flock, achieving an average accuracy of 99.7% and a running speed of 40 frames per second (fps). Fang et al. [16] used deep-neural-network (DNN)-based pose estimation to analyze broiler behavior for the first time, filling a gap in the use of skeletal feature points to calculate chicken behavior. Pu et al. [17] proposed a convolutional-neural-network (CNN)-based method while using Kinect sensors to identify chicken behavior with an accuracy of 99.17% for chicken lameness. All of the above research results demonstrate the accuracy and effectiveness of deep learning in the identification and detection of birds, which shows that deep learning has great value in poultry farming. However, very little research has been completed on ducks worldwide, and there is an urgent need to propose an efficient and accurate identification and detection method to promote the development of the duck-breeding industry.

This paper is divided into four sections, the present one being Section 1. In Section 2, the dataset is described, and the image pre-processing and accuracy evaluation methods are detailed. In Section 3, the experimental results and the training process of the Yolov5 model are presented and discussed. Furthermore, separate experimental comparative analyses of different classical classification network models and different processing methods are presented. Additionally, the applications and feasibility of the experiment are discussed. In Section 4, some salient features of the present study are highlighted.

## 2. Materials and Methods

### 2.1. Access to Materials

**Figure 2:**
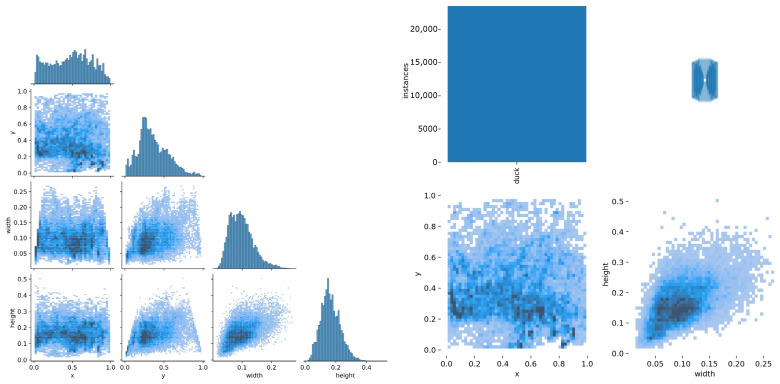
The distribution of ducks in the images of the dataset. x and y are the horizontal and vertical coordinates of the ducks located in the image. Width and height are the length and width of the ducks in the image. The distribution of the ducks’ positions follows a normal distribution in general.

The images used in this study were captured by DJI Pocket2 (Company: Tianjin, China, Tianjin Tongye Technology Co., Ltd. Headquarters: Shenzhen, China), a miniature gimbal camera that can be effectively and flexibly deployed in a variety of environments and can effectively meet the requirements of detection. The additional specifications of the DJI pocket2 are as follows: (1) frame rate: 24/25/30/48/50/60 fps; (2) color: black; (3) dimensions: 124.7 × 38.1 × 30 mm; (4) weight: 117 g; (5) electronic shutter speed: 8 s^−1^/8000 s; (6) lens equivalent focal length: 20 mm; (7) Gimbal maximum control speed: 120 °/s; (8) Gimbal jitter suppression: ±0.005°. All of the images were taken at the original waterfowl farm in Sichuan Province, China, and both the vertical resolution and horizontal resolution of the picture are 96 dpi, and the picture size is 518,400 pixels (960 × 540), which properly matches the angle range of the system’s surveillance cameras so that we could observe the ducks without any specific intervention on them. The shooting methods include long-distance shooting and short-distance shooting, and the shooting angles include top-down shooting and parallel shooting. Top-down and long-distance shooting occupies most of the dataset because this kind of shooting is more in line with the angle range of the system’s surveillance camera, which is more practical. We finished the recording in two days, and the dataset contains individual behaviors of the ducks, such as wing waving, which add to the richness and diversity of the data. In total, 500 ducks and 28 videos were used for the study, and the original dataset contains 1663 images. Additionally, images containing only one duck were further extracted from the detection results. The distribution of data is shown in Figure 2, x and y are the horizontal and vertical coordinates of the ducks located in the image, and width and height are the length and width of the ducks in the image. The distribution of the ducks’ positions follows a normal distribution in general, and the distribution of the dataset is close to the natural situation.

**Figure 3:**
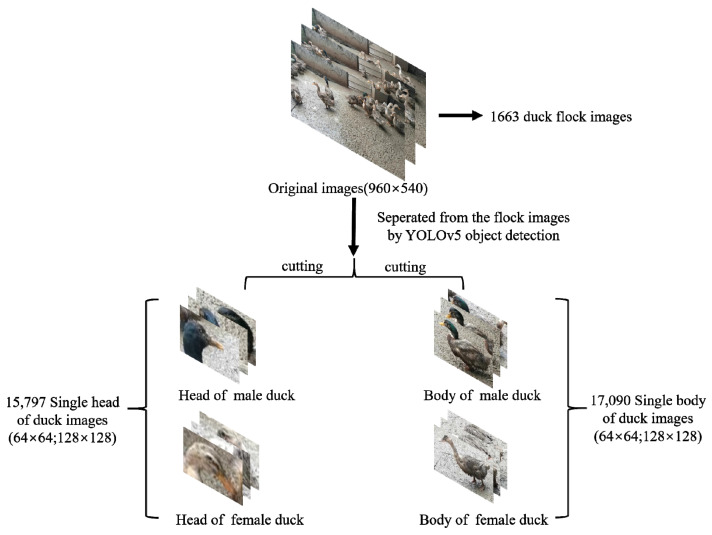
The original dataset consists of 1663 duck group images. The classification dataset of single duck images is extracted after Yolov5 target detection.

The position of the bounding box (both single-category and two-categories) for each duck in each image was marked by the researcher. The labeling of the bounding boxes follows the following principles: (1) the categories should be clear: for single-category target detection, the category is set to duck, and for two-categories target detection, the category is set to both male and female; (2) in the case where the target individual is obscured, truncated or blurred, the bounding box should be clearly defined: when the target individual is obscured or truncated, the bounding box should contain the key features of the target individual with no other individuals, and when the target individual is blurred, the sample is still involved in training to improve the robustness of the model; (3) boundary check after annotation: it is necessary to ensure that the bounding box coordinates are not on the image boundary to prevent out-of-bounds errors during data enhancement. The process of acquiring the dataset is shown in Figure 3:

### 2.2. Data Pre-Processing

#### 2.2.1. Mosaic Data Enhancement

**Figure 4:**
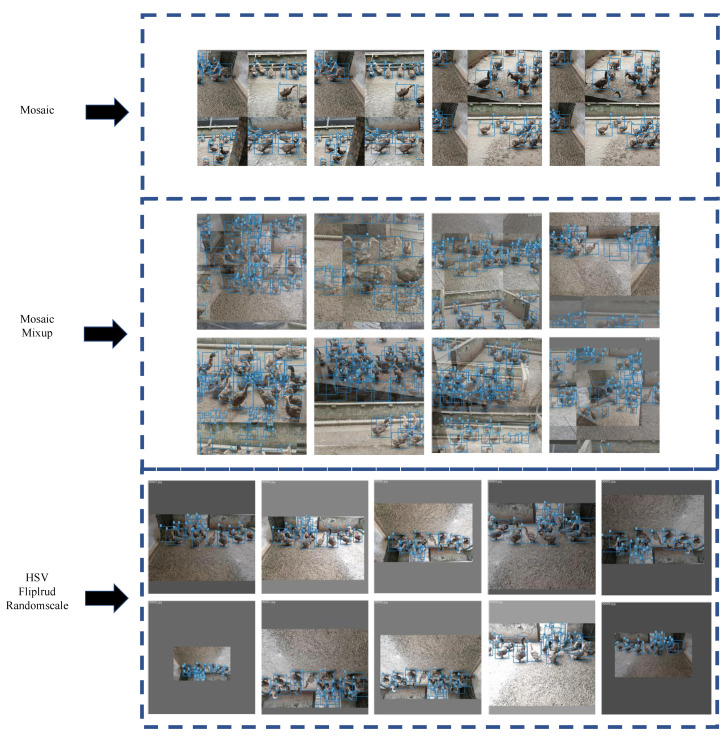
Images after data enhancement. (1) Mosaic: Images enhanced by Mosaic data enhancement. Four images are selected randomly, scaled and cropped randomly, and then stitched together in random order. (2) Mosaic and Mixup: Images after Mosaic and Mixup enhancement. First, any four images are scaled and stitched together. Next, two random samples are mixed proportionally, and the results of the classification are assigned proportionally. (3) HSV, Fliplrud, and Randomscale: Images after HSV, Fliplrud, Randomscale. Images are randomly flipped and scaled with a probability of 50%. At the same time, the hue, saturation, and value of the images are changed.

Mosaic data enhancement was proposed by Yolov4 [18], and the main idea is randomly scaling, cropping, and arranging four images and then stitching them into a single image as training data. The two main advantages are as follows: (1) enriching the dataset: randomly using four images, randomly scaling and cropping, and then randomly lining them up for stitching, enriching the detection dataset, where random scaling adds many small targets and improves model robustness; (2) reducing the video memory occupation: the data of four images are directly calculated during training, reducing the dependence on batch size, and even a single GPU can train Yolov4. The results are shown in Figure 4:

#### 2.2.2. Mixup

Data augmentation is a method of expanding the dataset and is divided into several independent augmentation processes (e.g., flipping, rotating, scaling, shifting, blurring, etc.) and mixed class augmentation process (e.g., Mixup) [19]. Mixup is an algorithm used in computer vision for mixing and enhancing images, which allows the mixing of images between different classes to construct new training samples and labels in a linear interpolation manner, thus expanding the training dataset. This paper follows the given setting of super parameters (alpha = beta = 0.5) [20]. Alpha and beta are the two parameters of the Beta distribution. The result of the mixup is shown in Figure 4.

#### 2.2.3. Other Tricks

Data enhancement changed the HSV of the original picture [21]. The operation includes three aspects: hue (H), saturation (S), and value (V). Fliplrud refers to the fliplr function and the flipud function, both of which are functions that operate on matrices. More specifically, the fliplr function implements the left and right flip of the matrix, and the flipud function implements the up and down flip of the matrix. Additionally, using the randomscale function, the scale of the image is randomly transformed according to a preset probability. The picture is randomly flipped and scaled with a probability of 50%. The images processed by the above method are shown in Figure 4.

### 2.3. Related Networks

#### 2.3.1. YOLOv5

YOLOv5 is a single-stage target-detection algorithm. For a target-detection algorithm, we can usually divide it into 4 generic modules, which include input, backbone, neck, and output. The input side represents the input image. This module usually consists of an image pre-processing phase. In the network training phase, YOLOv5 uses Mosaic data enhancement operations to improve the training speed of the model and the accuracy of the network and proposes an adaptive anchor frame calculation and adaptive image scaling method. The backbone networks are usually networks of some high-performance classifier species. YOLOv5 uses not only the CSPDarknet53 structure but also the Focus structure as the backbone network. The neck network is usually located in the middle of the backbone network and the output side; using it can further improve the diversity and robustness of the features. Although YOLOv5 uses the same SPP module and FPN + PAN module as YOLOv4, the implementation details are different. The output side is used to complete the output of the target-detection results. YOLOv5 uses the GIOU Loss function instead of the Smooth L1 Loss function to further improve the detection accuracy of the algorithm.

#### 2.3.2. VovNet_27slim

VoVNet [22] is a GPU computing-focused and energy-efficient network. The authors improve the dense_block in DenseNet from the perspective of MAC (memory access cost) and GPU parallel computation efficiency to obtain an OSA (one-shot aggregation) module that combines performance and efficiency. In the OSA module, each layer generates two kinds of connections, one to the next layer via conv to produce a feature map with a larger receptive field and the other to the final output layer to aggregate good enough features. Further, the authors built VoVNet_27slim, VoVNet_39, and VoVNet_57 based on the OSA module. VoVNet starts with a stem block of three 3 × 3 convolutional layers, followed by a 4-stage OSA module, with a 3 × 3 max pooling layer with a stride of 2 being used for downsampling at the end of each stage. VoVNet_27slim is a lightweight model, while VoVNet_39/57 contains more OSA modules at stage 4 and stage 5, so the model is larger. VoVNet offers a significant improvement over DenseNet at the same level in terms of computational speed and power consumption.

### 2.4. Detection and Evaluation Methods

#### 2.4.1. Detection Methods

This paper presents a computer-vision-based recognition detection algorithm for target detection and sex classification of ducks on farms, as well as an outlook for duck sex ratio estimation. Using this algorithm, breeders can obtain the duck sex ratio and achieve rapid management of the farm to optimize the reproductive rate and growth of the ducks, which helps to maximize economic efficiency. Given the small and dense individuals in the duck population, we choose to use the Yolov5 model with multi-scale predictive capabilities. The input side of Yolov5 uses Mosaic data augmentation, stitching by random scaling, random cropping, and random lining up, which is particularly suitable for small target detection [23].

To achieve the best detection results, four experimental protocols are proposed in this paper:

1. Scheme one: Target detection for the whole ducks using Yolov5 in the target-detection phase, where the classifier is set to two categories, female and male, to directly obtain the sex ratio of the ducks;
2. Scheme two: Target detection for duck heads uses Yolov5 in the target-detection phase, where the classifier is set to two categories, female and male, to directly obtain the sex ratio of the ducks;
3. Scheme three: In the target-detection stage, target detection is carried out using Yolov5 for duck heads, where the classifier sets only one category of the duck, and then the detected head pictures of a single duck are fed into the classification network model. Finally the classification network carries out sex classification to obtain the sex ratio of the ducks;
4. Scheme four: In the target-detection stage, target detection is carried out using Yolov5 for the whole ducks, where the classifier sets only one category of the duck, and then the detected body pictures of a single duck are fed into the classification network model. Finally the classification network carries out sex classification to obtain the sex ratio of the ducks.

**Figure 5:**
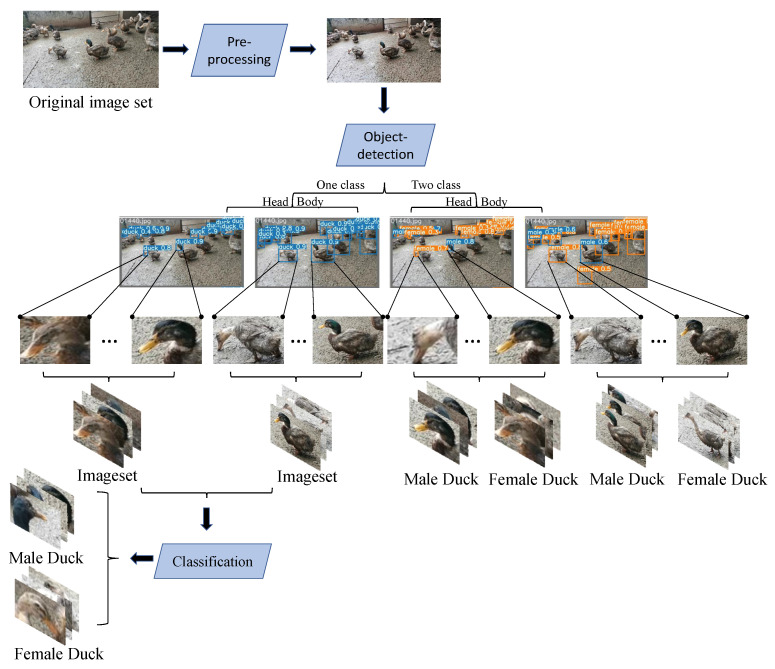
The general flow of the four schemes. The original image is pre-processed and fed into the target-detection network. When the image contains only one label, the target detection network only detects the duck category and then extracts the head and body of the duck from the completed image, and sends them to the classification network, respectively, to complete the recognition task. When the image contains two labels, the target-detection network detects both the male and female categories to complete the recognition task.

The overall flow of the proposed schemes is shown in Figure 5. The original dataset is pre-processed, and the target detection is performed for the whole body or the head of the duck in either two categories or a single category. The former is to obtain the duck’s sex ratio directly after the two categories’ target detection. In the latter method, after the single-category target detection, the whole body image of a single duck or the head image of a single duck is passed through the classification network so that we can obtain the sex ratio of the duck as well.

In fact, the above four schemes constitute a set of comparison experiments, with the variables being the number of detection categories of the target-detection network and the type of input image of the classification network. Further, within each scheme, there are also comparison experiments with variables such as the type of the target-detection network and classification network and the input image size of the classification network. The various comparison experiments enable the optimal model to be found efficiently.

#### 2.4.2. Focal Loss

**Figure 6:**
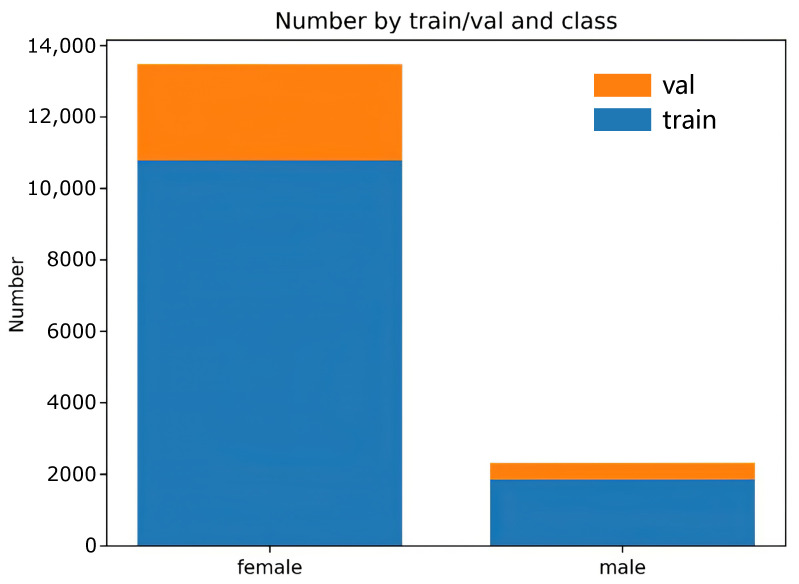
The average sex ratio of ducks in the population images.

Category imbalance is a situation where the number of training samples differs significantly between categories in a classification task, which can cause problems for the learning process [24], and Leevy et al. [25] describe many existing solutions. Focal loss is an improved cross-entropy loss function that can solve the problem of severe imbalance in the proportion of positive and negative samples in one-stage target detection and achieve high accuracy in a one-stage structure [26]. In the population images of the dataset presented here, the sex ratio of ducks is concentrated at 1:7 (males:females), with an imbalance between positive and negative samples. Therefore, this paper uses Focal loss as the loss function. The average sex ratio of ducks in the population images is shown in Figure 6. The blue part of the figure represents the number of training sets, and the orange part represents the number of validation sets, divided by a ratio of 8:2.

#### 2.4.3. IoU, Accuracy, Precision, Recall, and F1 Score

Intersection over Union (IoU) [27] is the intersection ratio between the model’s predicted bounding box and the true bounding box and is a common metric used to evaluate the accuracy of target-detection algorithms. The IoU is calculated by Equation (1).

(1) $$\mathrm{IoU}=\frac{A\cap B}{A\cup B}$$

In it, A and B refer to the bounding box and the ground truth box in the target detection.

Accuracy refers to the ratio of the number of correctly classified samples to the total number of samples and is a commonly used assessment metric. The following Equation (2) calculates the accuracy.

(2) $$\mathrm{accuracy}=\frac{\mathrm{TP}+\mathrm{TN}}{\mathrm{TP}+\mathrm{TN}+\mathrm{FP}+\mathrm{FN}}$$

In it, T and F refer to True and False, representing whether the prediction is correct or not, and P and N refer to Positive and Negative, representing the category of the prediction. TP (True Positive) stands for predicting the positive class as positive, FN (False Negative) stands for predicting the positive class as negative, FP (False Positive) stands for predicting the negative class as positive, and TN (True Negative) stands for predicting the negative class as negative.

Precision refers to the proportion of samples correctly judged to be in a positive class out of those predicted to be in a positive class, which is a common assessment metric used in binary classification problems. Equation (3) listed below calculates precision.

(3) $$\mathrm{precision}=\frac{\mathrm{TP}}{\mathrm{TP}+\mathrm{FP}}$$

Recall refers to the proportion of samples that are correctly judged to be in a positive class out of those that are actually in a positive class, which is a commonly used assessment metric in binary classification problems. Equation (4) listed below calculates recall.

(4) $$\mathrm{recall}=\frac{\mathrm{TP}}{\mathrm{TP}+\mathrm{FN}}$$

Since precision and recall are contradictory metrics, we adopt the F1 score. The formula for the F1 score is simplified when both precision and recall weights are one. In Formula (5), P stands for precision, and R stands for recall. Therefore, we can calculate the F1 score from Equation (5).

(5) $$F_{1}=\frac{2\mathrm{PR}}{P+R}=\frac{2\mathrm{TP}}{2\mathrm{TP}+\mathrm{FP}+\mathrm{FN}}$$

#### 2.4.4. Average Precision

Average precision is a model evaluation metric widely used in target-detection papers. The original AP calculation method for VOC [28] was changed in 2010. The AP calculation method changed after 2010 [29] is now commonly used and is also more reasonable than the previous method. Based on these two calculations, assuming that there are M positive cases in N samples, M recalls are obtained. For each recall value r, the maximum precision corresponding to (r’ > r) is calculated, and then the AP is obtained by averaging these M precisions. The PR curve is calculated by Equation (6).

(6) $$\mathrm{AP}=\int_{0}^{1}p\left(r\right)\mathrm{dr}$$

Generally speaking, AP is the mean value under a single category, and Mean Average Precision (mAP) is the mean value of AP under all categories. Equation (7) is used for calculating mAP.

(7) $$\mathrm{mAP}=\frac{\sum_{q=1}^{Q}\mathrm{AP}\left(q\right)}{Q}$$

In this equation, Q is the number of all categories, and the denominator is the sum of the AP of all categories.

### 2.5. Experimental Environment

The server platform is configured with Intel(R) Xeon(R) Gold 5218 CPU @2.30GHz, 128 GB RAM, 2 TB hard disk capacity, 2 × RTX 5000 GPUs (32 GB total video memory), and Ubuntu 18.04 operating system.

### 2.6. Training Strategies

**Figure 7:**
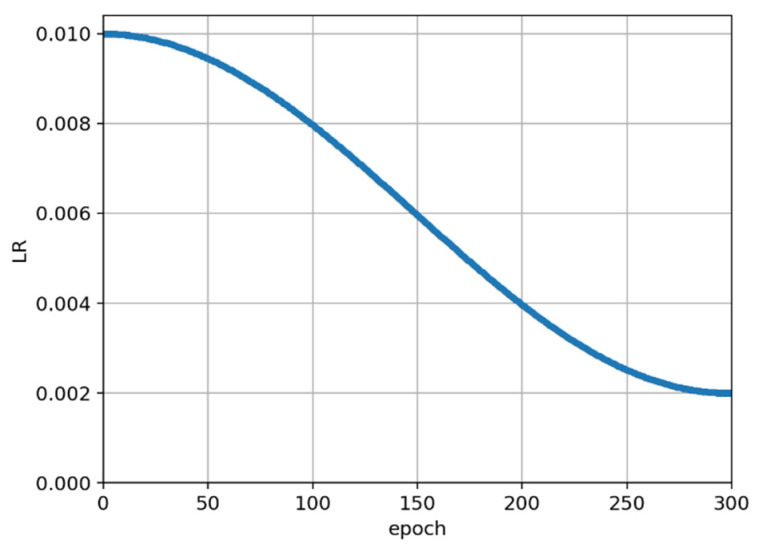
Learning rate strategy for training.

In the training process of the experiment, the SGD optimizer is used, and the initial learning rate is set to 0.01. The momentum and weight decay are, respectively, set as 0.9 and 0.0005. Nesterov is set to True, i.e., Nesterov momentum is introduced in stochastic gradient descent. For the model with target detection, the batch size is set to 16, and the training image size is 409,600 pixels (640 × 640). For the model of the classification network, the batch size is set to 256; also, the image size of the whole duck and the head of the duck is much smaller than the original size, and the training image size is 4096 pixels (64 × 64). The learning rate strategy for training is shown in Figure 7, using Cosine learning. The Cosine learning rate decay curve is relatively smooth, which can effectively improve the training accuracy of the experiment. There were 300 epochs in total, and the learning rate gradually decreased from 0.01 to 0.002.

## 3. Results

### 3.1. General Description

**Figure 8:**
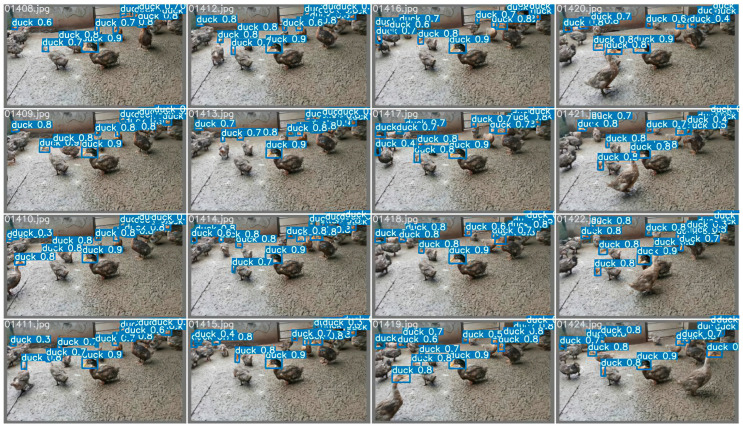
The effect of the hemp duck sex estimation model. The locations, masking conditions, and individual behaviors of the ducks vary in each extracted frame. In the final model, the accuracy reached 99.29%, F1 score reached 98.60%, and fps reached 269.68.

**Figure 9:**
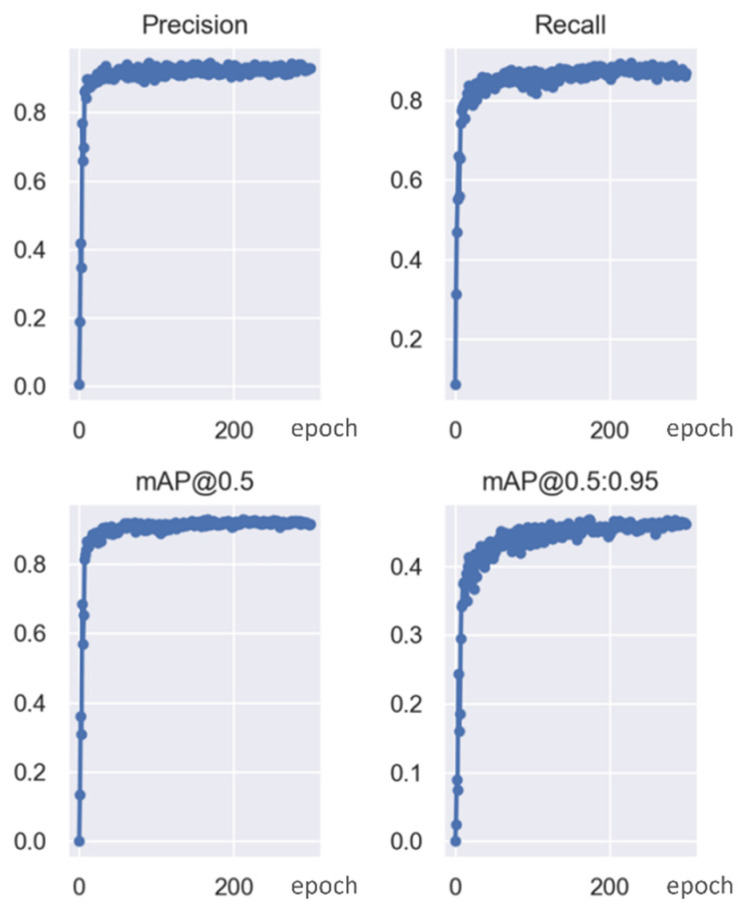
Precision, recall, mAP changes, and convergence process during training.

**Figure 10:**
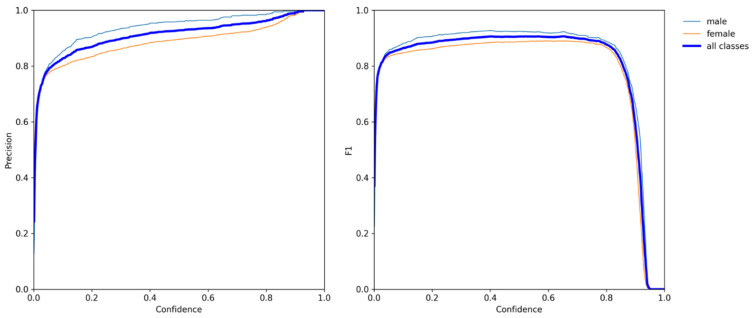
The relationship between precision, F1 score, and confidence of the model in classifying the ducks as male and female.

Yolov5 and VovNet_27slim combined models realized the sex estimation of ducks using head color as the main criterion. In the final model, the accuracy reached 99.29%, the F1 score reached 98.60%, and fps reached 269.68. For the classification network, the input images were filtered by the Yolov5 detector in advance and obtained 15,797 images. A classification dataset of a single head of duck images was obtained, and then a variety of different classification networks were trained and validated. In this process, the pre-training mode was used for transfer learning. The result of the final model is shown in Figure 8 listed below; the training process is shown in Figure 9 and Figure 10 listed below.

In this experiment, the following results can be obtained:

1. Through comparative experiments, the optimal scheme of the Yolov5 and VovNet_27slim combination is obtained;
2. The duck-head detection experiment of the duck group was completed by the Yolov5 detector, and by applying the results from the above experiments, a large number of images of ducks’ single heads were cropped, then a single-head dataset containing 15,797 images was generated by manual screening;
3. The whole-duck detection process was completed by the Yolov5 detector, and the above experiments were used to obtain a large number of completely framed duck images; then, the whole-duck dataset containing 17,090 images was generated by manually labeling the sex of the ducks;
4. The performance of the major classification networks in the sex dichotomies of ducks was compared, and the use of the VovNet_27slim network was determined;
5. In the ablation experiment of the Yolov5 detection, HSV_Aug, Mosaic, and MixUp were added to improve the evaluation index, thus enhancing the effect of the model;
6. The combined model of the Yolov5 and VovNet_27slim was used to estimate the sex of the duck;
7. The superiority of single classification object detection and dichotomous classification network in the sex estimation of ducks was proven by comparison; also, it provides a novel solution for other classification problems based on local characteristics.

At the same time, this experiment compares the four schemes mentioned above to achieve the sex estimation of ducks. The specific flow chart is shown in Figure 5.

### 3.2. Object Detector

**Figure 11:**
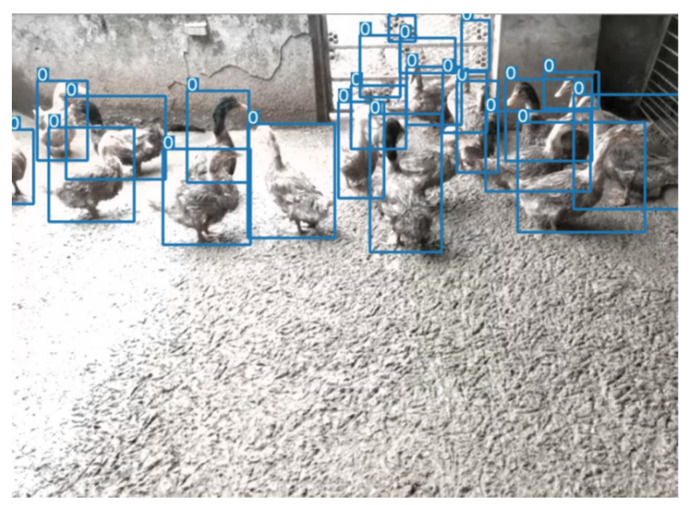
The effect of the Yolov5 detector (without tricks). The 0 in the image represents the single category detected and the blue rectangular in the image represents the target detection frame. Because this is a detector comparison experiment, the category information for the detected objects in the image is not labelled.

As can be seen from Figure 2, all four schemes need to be processed by the object detector. The main target-detection models are compared on a single-head dataset and evaluated the precision, recall, and other metrics one by one. Finally, Yolov5 is selected as the final object detector. The comparison of object detectors is shown in Table 1 and Table 2. The effect of the Yolov5 detector is shown in Figure 11. As can be seen from Table 1, Yolov5 has a considerable advantage over other detectors in speed in the case of high accuracy. Although Yolov5 is used as a single classification detector in some schemes, it is still the best choice in terms of overall effect and speed. Therefore, the object detector used in all schemes in this paper is Yolov5.

**Figure 12:**
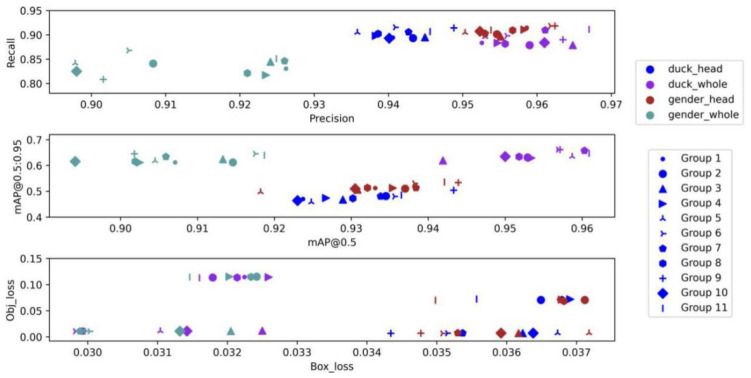
Ablation experiment corresponding to the four schemes, with each point corresponding to a group of tricks, in which each color represents each scheme and the shape of each point represents different experimental groups. The comparison shows that when focal loss calculation is introduced and HSV_Aug, Mosaic, MixUp, Fliplrud, and RandomScale are used, there is a degree of improvement in mAP, although the Yolov5 model does not have a significant advantage in terms of precision and recall compared to the other combinations. The results show that group 11 is the best.

Based on Yolov5, some tricks were tried in the ablation experiment. By combining and splitting different tricks and then comparing the effects through training and verification, Table 3 and Figure 12 below were obtained. Through comparison, it can be seen that when the focal loss is introduced to calculate loss and HSV_Aug, Mosaic, MixUp, Fliplrud, and RandomScale are used at the same time, although the Yolov5 model has no obvious advantages in precision and recall compared with other combinations, mAP has a certain degree of improvement. The results show that the 11th group is the best. Additionally, the improvement in mAP undoubtedly reduces the difficulty of the classification network and has a positive impact on the improvement of the whole model’s capability. Specific indicator data can be found in Table 3.

For the object detector, the following conclusions were obtained through experiments:

1. According to the comparison between Yolov5 and other detectors in Table 1, Yolov5 is the detector with the best comprehensive ability in this experiment, with a high accuracy of 0.93 mAP and the fastest speed of verifying a picture in 8 ms;
2. According to the comparison of Group 1, 2, and 4 in Table 3 and Figure 12, HSV_Aug, Mosaic, and other tricks can make the precision and recall of the detector rise slightly but have no obvious influence on mAP. As can be seen from the comparison of groups 7 and 9, it is the introduction of focal loss that shows an obvious upward trend in the model effect. By reducing the weight of samples that are easy to classify, this function makes the model focus more on samples that are difficult to classify during training, thus improving the overall effect.

The next four sections focus on the above four schemes:

### 3.3. Scheme One

**Figure 13:**
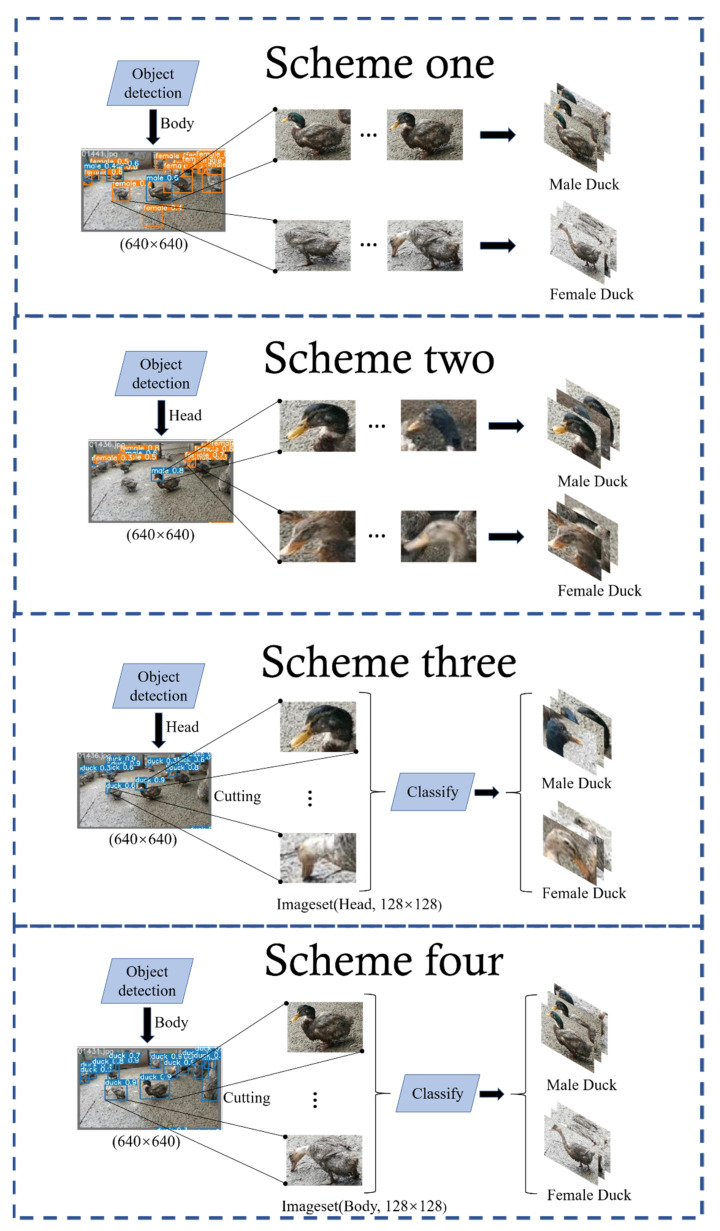
Flow chart of four schemes.

In this section, the flow chart of scheme one is shown in Figure 13, the image of the whole duck is directly processed by dichotomous Yolov5, and the individual duck is selected in the box and dichotomized by sex.

**Figure 14:**
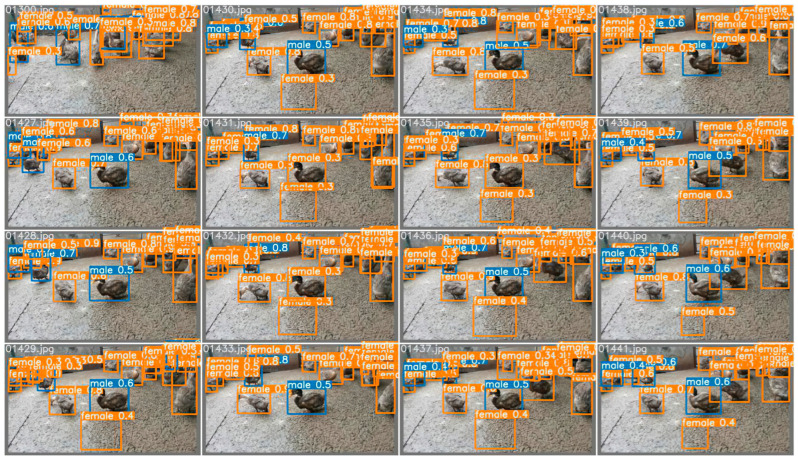
The sample results of scheme one. The locations, masking conditions, and individual behaviors of the ducks vary in each extracted frame. As can be seen from the final validation map, some of the ducks were not identified, and some of those that were identified were incorrectly estimated.

The whole-duck dataset mentioned above contains a large number of duck images framed in standard boxes with sex labeling. The whole-duck dataset was used for the training and verification of dichotomous Yolov5. The final verification figure is shown in Figure 14 below, in which parts of the ducks were not identified, and a considerable number of the identified ducks were incorrectly estimated.

**Figure 15:**
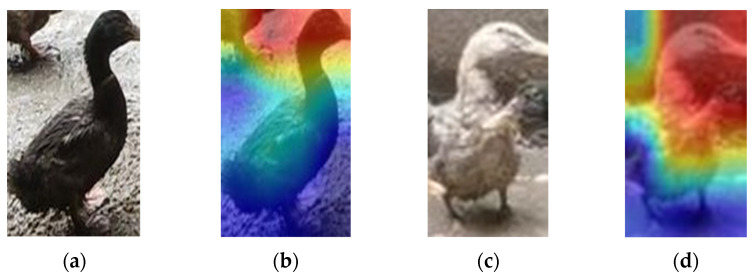
Heat map of VovNet_27slim. (**a**,**b**), respectively, show the original and heat map of the male duck; (**c**,**d**), respectively, show the original and heat map of the female duck. The redder the color, the more interested the computer is in the area, and the bluer the color, the less concerned it is. Grad-CAM shows that the network focuses on the heads of male and female ducks.

To better understand the classification network model and visually explain it, Grad-CAM [30] is generated from the input image of a duck (including whole body and head images), as shown in Figure 15. VovNet_27slim was chosen as the visualization model because the accuracy and speed of VovNet_27slim’s classification of the sex of the ducks reached a very high level, which will be explained in more detail below. Grad-CAM showed that VovNet_27slim focused on the same part of the body in most cases, with the model focusing on the head in both male and female ducks. Therefore, this model mainly classifies ducks according to their heads.

It can be seen in Figure 15 that the head of the duck is the main feature that the computer uses to determine the sex of the duck. The redder the color, the more interested the computer is in the area, and the bluer the color, the less it cares. However, when the whole duck was detected in this section, the factors interfering with the detector’s judgment increased; the color of the body parts and the difference in duck shape may become the noise points in the object detection network. Moreover, object detection is not strong enough to deal with the multi-classification problem, which leads to the poor effect of sex estimation in the final experiment. For these two reasons, in the following sections, experiments are carried out to prove the correctness of the above analysis.

To summarize, this section has strong limitations in dealing with gender estimation, and the verification effect is poor, so this scheme was not selected in the end.

### 3.4. Scheme Two

In this section, the specific experimental process of scheme two is shown in Figure 13. The whole-duck dataset used for object detector training is replaced by the single-head dataset of the duck to try to solve the problem of extra noise points introduced as a result of selecting the whole duck in the frame mentioned above. The object detector remains unchanged and is still dichotomous Yolov5.

**Figure 16:**
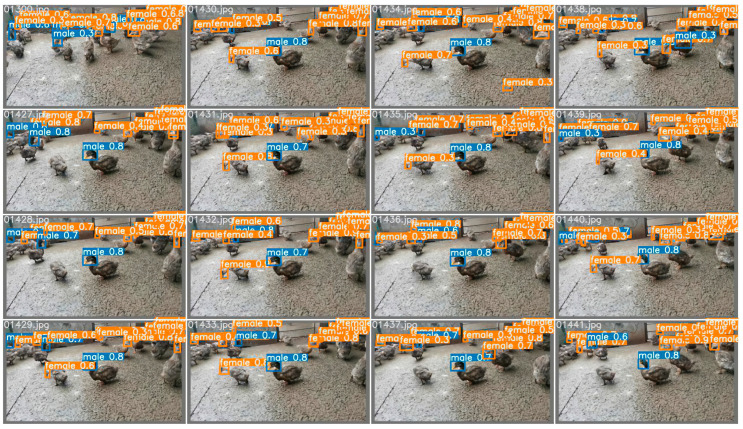
The sample results of scheme two. The locations, masking conditions, and individual behaviors of the ducks vary in each extracted frame. Change to the training dataset reduces the number of additional noise points and slightly improves the success of duck detection and the accuracy of gender estimation, but the accuracy is still not high.

The single-head dataset of ducks used in this section contains a large number of images of a single head of ducks with sex labeling. Similarly, a single-head dataset of ducks was used for the training and verification of Yolov5, and the verification effect is shown in Figure 16 below. It can be seen that by changing the training dataset, the reduction in extra noise points slightly improves the detection success rate of the ducks, and the accuracy rate of sex estimation also increases, but the accuracy is still not high. The following sections try to improve the accuracy of sex estimation by adding classification networks to the model.

Although this section reduces the extra noise point by focusing on local characteristics and, to some extent, enhances the ability to solve the problem of sex estimation, it still does not have high precision, which suggests that a single-object-detection model cannot make up for the defects in classification problems, as expanding the dataset is unable to solve the problem that stems from the source. Therefore, the scheme in this section is not selected, and the following sections continue to discuss and study this issue.

### 3.5. Scheme Three

The flow chart of scheme three is shown in Figure 13: to solve the problem of low accuracy of sex classification caused by only using the object detector, the dichotomous object detector was changed into a single one based on experiment 3.2 and added the classification network later to solve the problem of sex classification.

Considering that this section needs to use the classification network to deal with the dichotomy of the sex, the single-head image output was re-screened by the target detector, selected 15,797 images for uniform size, and then sent them to the classification network for training. Similar to the object detector, comparative experiments of various classification networks were carried out, and the specific comparison is shown in Table 4. The accuracy of the VovNet_27slim model is only above average among many models, but its F1 score is the highest, and it has advantages in speed and fps. After comparing with the actual verification effect diagrams of other models, VovNet_27slim was finally selected as the classification network.

Through comparative experiments, the importance of input picture size in the training process of the classification network is analyzed. In the above experiments, the VovNet_27slim model was determined as the final classification network, and we compared and verified the models with input sizes of 4096 pixels (64 × 64) and 16,384 pixels (128 × 128), respectively. The detailed evaluation metrics and comparison are shown in Table 5 below. Although the model with an input size of 16,384 pixels (128 × 128) has some disadvantages in terms of time and fps, it takes the lead in all metrics related to accuracy. In addition, Yolov5, used before the classification network, already has an extremely fast speed, so accuracy should be pursued more in the classification network. Overall, a model with an input size of 16,384 pixels (128 × 128) was chosen.

Similarly, the following conclusions can be drawn from this section:

1. With the deepening of the classification network structure of the ResNet series, the overall speed and fps tend to slow down; also, the accuracy and F1 score decrease, and the overall effect gradually declines;
2. The MobileNet series, which is famous for being lightweight, has basically similar effects but has no obvious advantages compared with other models. The advantages of speed and fps are not reflected in the experiment, and the overall effect is not ideal, which also indicates that it cannot fully exploit its advantages in the small dataset;
3. VovNet_27slim relies on its OSA module and optimizes its network structure to be very lightweight by performing a downsampling operation at the end of each stage. In comparison experiments, the advantages of its speed and fps were fully demonstrated. At the same time, due to its inheritance of DenseNet advantages, the reasoning ability of the model was also improved.

**Figure 17:**
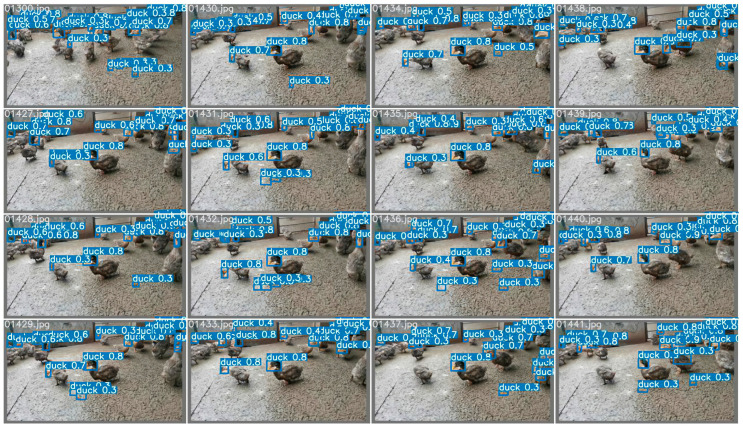
The effect of scheme three. The locations, masking conditions, and individual behaviors of the ducks vary in each extracted frame. The single-head dataset of the ducks improves duck detection success and sex-estimation accuracy.

In this section, the classification network is introduced, and the single-head dataset of the ducks is used to improve the detection success rate and sex estimation accuracy of the ducks. The specific effects are shown in Figure 17 below:

### 3.6. Scheme Four

To prove the advantages of using a single-head dataset of ducks, this section is used for comparison. The specific experimental process of scheme four is shown in Figure 13. Aiming at the defects of Yolov5 in classification proposed in Section 3.2, the classification of the Yolov5 detector is changed from binary to one-class, and the classification network is added after the detector to solve the sex-estimation problem. This section aims to verify the superiority of the single-head dataset by comparing it with Section 3.3.

**Figure 18:**
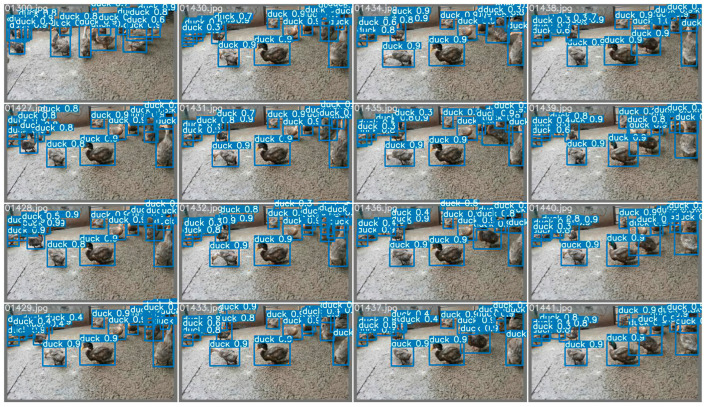
The effect of scheme four. The locations, masking conditions, and individual behaviors of the ducks vary in each extracted frame. The accuracy of sex estimation for detected ducks improved considerably compared to scheme one and scheme two but still does not exceed the results of scheme three.

In this section, a classification network is introduced after an object detector to improve the disadvantage of an object detector in classification. In terms of the dataset, the whole-duck dataset of ducks is still used, which is compared with Section 3.1 to verify the positive impact of joining the classification network and compared with Section 3.3 to verify the advantages of a single-head dataset. Similarly, the whole-duck dataset was used for training and verification, and the final effect is shown in Figure 18 below. As can be seen from Figure 18, the accuracy of sex estimation for detected ducks was greatly improved compared with Section 3.1 and Section 3.2, but the effect of Section 3.3 could not be exceeded. In addition, in a detailed comparison with Section 3.3, we can see that there are still some problems that result in the unsuccessful determination of the sex of the ducks. These problems are caused by the existence of extra noise points mentioned above, which affect the ability of the model to make judgments. In summary, this section was not selected.

Through a comparison of the previous four sections, it is easy to see that in Section 3.3, based on using the single-head dataset of ducks as the training set, a classification network was also introduced, and a screening of the classification network was carried out. Finally, the model effect obtained was significantly improved compared with the other three groups. To summarize, the scheme described in Section 3.3 is optimal and was selected as the final scheme.

In addition, to achieve the best effect of the model, the duck farm has the following requirements in practical application scenarios:

1. The density in the activity area of ducks is low, and there is no overlap of ducks;
2. In the sampling area of the duck farm, there should only be ducks and not other animals, especially birds. The dataset used for training in this experiment is only for ducks. If there are other birds, the results will be interfered with, and subsequent management will not be conducive.

## 4. Discussion

Sex classification is a branch of target detection that has great potential for application in livestock rearing. Data from semi-free-range farms differ from previous datasets because of their relatively large randomness and irregularity. This paper proposes a two-stage method for estimating the sex ratio of hemp ducks based on the effect of the sex ratio on economic efficiency and explores the performance of target detection and classification networks for detection and sex classification. Based on this, the following topics are discussed.

### 4.1. Contribution to Sex Estimation in Poultry

Sex classification for poultry differs from general target-detection applications in that poultry targets are small, densely distributed, and have subtle features that distinguish sexes, features that make conventional target-detection networks inappropriate for semi-free-range poultry. In this study, we use a deep-learning-based approach for duck detection and sex classification and propose a high-precision algorithm for duck detection and sex classification. First, we experimentally analyzed the accuracy of classifying ducks based on detecting their bodies and heads and then classifying them based on the detected bodies or heads. The experimental results show that the Yolov5 object detector and the VovNet_27slim classification network have the best performance, with 99.29% accuracy, 98.60% F1 score, and 269.68 fps. Therefore, the two-stage model proposed in this paper can extract sex features very well, which can also be confirmed from the feature heat map. Finally, the model is evaluated, and the speed of the model meets the requirements of practical applications. Based on the experimental results, the implementation of automatic duck sex ratio estimation is discussed, providing insights into the automatic management of farms. Currently, there are few studies on automatic sex ratio estimation methods for farming, so this study fills this gap to some extent.

### 4.2. The Effectiveness of the Proposed Method

A total of four schemes were designed in this study. The first two schemes directly used the target-detection network to detect and classify the whole body and head of the duck, respectively, by gender, while the latter two schemes first used the target-detection network to detect and single-classify the whole body and head of the duck, respectively, and then used the classification network to classify the whole body and head of the detected duck by gender, respectively. As the single-stage model (e.g., Yolov4) with classification, box, and confidence all based on a shared feature map does not decouple this information well, we believe there is a risk that this information may interfere with each other, resulting in poor extraction of gender-feature information. Therefore, we use a two-stage model with the aim of decoupling these features, so that object detection and classification do not interfere with each other. The two-stage approach based on Yolov5 and VovNet_27slim proved to be very effective. Among them, Yolov5 has a strong detection capability, and VovNet_27slim has a strong classification capability. VovNet_27slim is inherited from DenseNet and constituted by the OSA module. In terms of effect, it has a stronger model ability than the major classic-object detection backbone, ResNet. In terms of speed, it also has a great advantage, and its high fps indicates that it has a strong real-time detection ability. In future work, we will consider integrating it with PlantNet [31] modules for deployment. Furthermore, under the condition of the same or even worse hardware, it can also maintain a relatively high-level detection ability and processing speed. Moreover, Grad-CAM verified that feature extraction was focused on the head of the duck.

In addition, in the process of original image collection, although this research used camera equipment for sampling, the image of the duck was collected from top to bottom in different positions from different angles, the monitoring camera equipment was simulated reasonably, the obtained information was more diverse, and the information collection of the ducks was more accurate in various situations. Although multi-angle shooting increases the noise points that have negative effects on the model, compared with single-angle sampling, the frequency of the same noise points was greatly reduced in the training image, thereby reducing their weight in the final image. In addition, the diversity and quantity of effective information can not only increase the weight of key feature information in the model but also improve the robustness of the model and give the model a stronger generalization ability. In fact, the increase in interference information brought by multi-angle shooting indeed makes the model ignore a small part of the effective information of the edge, but when the number of images collected reaches a certain amount, the loss of individual effective information does not cause the change of the overall structure (sex ratio).

Therefore, the method proposed in this study is effective and feasible. It has certain reference value and practical significance in solving the problem that takes local features as the main classification criterion and offers a new solution for this kind of problem.

### 4.3. Limitations and Future Work

It must not be overlooked that this study still has limitations.

Firstly, this study does not take into account disturbances caused by the external environment, such as different species of ducks on the farm or birds in the field environment. It is worth noting that if the farm is a mixed free-range farm, then computer-vision-based poultry image recognition is a fine-grained image-recognition task dedicated to distinguishing subclasses under a broad category, and research in this area could provide ideas to address this problem. For example, Zhi Xuye [32] used a part-based idea to divide the recognition task into four modules: target and part detection, data augmentation, feature extraction, and classification decision, and the proposed algorithm could achieve 89.7% recognition accuracy on the validation set of the CUB200-2011 database. This performance metric is among the most advanced available.

Next, there are false detections and missed detections in the detection results. For false detection, GAN [33] is used to generate high-resolution images or high-resolution features while finding a more appropriate confidence level. For missed detection, this study tries to improve the performance of small target detection by using Soft-NMS [34] or by fusing different downsampled feature layers.

In addition, the detection results are greatly affected by the overdetection. There are three main solution ideas. Firstly, we can improve the Non-Maximum Suppression [35] method to find the best target bounding box and eliminate the redundant bounding boxes. Secondly, a rectangular oblique box labeling approach [36] is used. This approach has no redundancy and only one numerical representation of the same range box, which is advantageous for the detection of targets with arbitrary orientation and dense arrangement. Thirdly, an instance segmentation approach is used to accurately extract individuals for classification. This approach can largely eliminate redundant feature information but inevitably has an increase in time complexity.

In summary, this study attempts to explore different algorithms to determine the best algorithm for the case of duck flock uncertainty and to deploy a network model to hardware devices for practical application on farms.

## 5. Conclusions

In this paper, a large-scale dataset for sex classification of hemp ducks is built, including 1663 duck images, 17,090 full-body images of individual ducks, and 15,797 head images of individual ducks, which can provide data support for future research on poultry. A two-stage method for estimating the sex ratio of hemp ducks is proposed, in which the duck heads in the input images are first detected and clipped by an object detector, and then the sex is judged by a classification network. Compared with a single-stage method, a better detection and recognition result was achieved. This study can provide a reference for future detection and recognition tasks for poultry and also provide inspiration for automated farming. In the future, this study will explore more effective algorithms to address the limitations that exist at this stage, including false detection, missed detection and surplus detection, and the deployment of network models to hardware devices.

## Figures and Tables

**Table 1:** Comparison of object detectors in a single-head dataset.

Model	Class	Precision	Recall	F1 Score	mAP@0.5	Inference Time @batch_size 1 (ms)
CenterNet	All	0.95	0.83	0.89	0.91	32
	Male	0.96	0.90	0.93	0.94	
	Female	0.94	0.76	0.84	0.88	
Yolov4s	All	0.80	0.91	0.85	0.91	10
	Male	0.81	0.93	0.86	0.93	
	Female	0.90	0.90	0.90	0.88	
Yolov5s	All	0.95	0.90	0.92	0.93	8
	Male	0.98	0.90	0.94	0.95	
	Female	0.92	0.89	0.90	0.90	
YoloR	All	0.82	0.91	0.88	0.93	11
	Male	0.89	0.92	0.90	0.94	
	Female	0.76	0.91	0.85	0.91	

**Table 2:** Comparison of object detectors in the whole-duck dataset.

Model	Class	Precision	Recall	F1 Score	mAP@0.5	Inference Time @batch_size 1 (ms)
CenterNet	All	0.99	0.58	0.73	0.91	32
	Male	0.98	0.60	0.75	0.91	
	Female	0.99	0.55	0.71	0.91	
Yolov4s	All	0.70	0.85	0.77	0.86	10
	Male	0.69	0.85	0.76	0.87	
	Female	0.72	0.85	0.78	0.86	
Yolov5s	All	0.89	0.83	0.86	0.91	8
	Male	0.93	0.80	0.86	0.92	
	Female	0.85	0.86	0.85	0.91	
YoloR	All	0.76	0.83	0.80	0.90	11
	Male	0.75	0.78	0.76	0.87	
	Female	0.78	0.89	0.83	0.93	

**Table 3:** In ablation experiment of Yolov5, each group of experiments corresponds to a group of tricks and evaluation metrics. The “×” means that the trick is not used and the “√” means that the trick is used.

Group	HSV_Aug	FocalLoss	Mosaic	MixUp	Other Tricks	Precision	Recall	F1 Score	mAP@0.5	mAP@0.5:0.95
1	×	×	×	×	×	0.93	0.87	0.90	0.91	0.45
2	√	×	×	×	×	0.91	0.88	0.90	0.90	0.46
3	√	√	×	×	×	0.92	0.89	0.91	0.91	0.44
4	√	√	√	×	×	0.93	0.90	0.91	0.92	0.45
5	√	√	√	√	×	0.92	0.89	0.90	0.91	0.46
6	√	×	×	√	×	0.92	0.89	0.91	0.92	0.46
7	√	√	√	×	Fliplrud	0.92	0.91	0.91	0.93	0.47
8	√	√	×	×	Fliplrud	0.92	0.88	0.90	0.91	0.45
9	√	×	√	√	Fliplrud	0.92	0.89	0.91	0.91	0.47
10	√	×	×	×	Fliplrud RandomScale (0.5~1.5)	0.93	0.88	0.91	0.92	0.47
11	√	√	√	√	Fliplrud RandomScale (0.5~1.5)	0.93	0.87	0.90	0.92	0.46

**Table 4:** Comparison of classification networks (input size is 4096 pixels (64 × 64)).

Model	Accuracy	Precision	Recall	F1 Score	Time (ms)	fps
ResNet_18	98.75	96.92	98.20	97.55	9	297.23
ResNet_34	98.98	97.89	98.05	97.97	9	167.63
ResNet_50	98.21	95.53	97.54	96.50	10	127.01
ResNext_50-32×4d	98.65	96.65	98.06	97.34	12	96.10
RegNetx_200mf	98.15	96.07	96.60	96.33	9	127.51
RegNety_200mf	98.42	97.01	96.69	96.84	10	96.12
MobileNetv2_1.0	98.83	97.51	97.83	97.67	9	153.09
MobileNetv3_small	98.83	97.23	98.15	97.69	8	173.50
MobileNetv3_large	98.88	97.39	98.18	97.78	9	142.21
GhostNet_1.0	98.51	96.51	97.63	97.06	9	79.50
ShuffleNetv2_1.0	98.24	95.39	97.88	96.58	9	130.96
DenseNet_121	99.00	97.32	98.79	98.04	11	55.88
VovNet_27slim	98.89	97.05	98.62	97.81	9	273.27
se-ResNet_18	99.02	97.92	98.18	98.05	9	232.09
se-ResNet_34	99.06	97.70	98.612	98.15	9	125.24
se-ResNet_50	98.43	96.51	97.30	96.90	11	95.80

**Table 5:** Contrast of input sizes of 16,384 pixels (128 × 128) and 4096 pixels (64 × 64).

Input Size	Accuracy	Precision	Recall	F1 Score	Time (ms)	fps
64 × 64	98.89	97.05	98.62	97.81	9	273.27
128 × 128	99.29	98.16	99.05	98.60	19	269.68

## Data Availability

Inquiries regarding the data can be directed to the corresponding author.
